# Evaluation of the impact of the Accelerate Pheno™ system on time to result for differing antimicrobial stewardship intervention models in patients with gram-negative bloodstream infections

**DOI:** 10.1186/s12879-019-4591-1

**Published:** 2019-11-07

**Authors:** Gerald Elliott, Michael Malczynski, Viktorjia O. Barr, Doaa Aljefri, David Martin, Sarah Sutton, Teresa R. Zembower, Michael Postelnick, Chao Qi

**Affiliations:** 1 Department of Pharmacy, Northwestern Memorial Hospital, Chicago, IL USA; 2 Northwestern Memorial Healthcare, 251 E. Huron St, Chicago, IL 60611 USA; 3 Clinical Microbiology Laboratory, Department of Pathology, Northwestern Memorial Hospital, Chicago, IL USA; 4 Department of Pharmacy Practice, Rosalind Franklin University, North Chicago, IL USA; 5 Department of Pharmacy, King Abdul Aziz Medical City, Jeddah, Saudi Arabia; 6 GlaxoSmithKline, Parsippany, NJ USA; 7 Division of Infectious Diseases, Department of Medicine, Northwestern University Feinberg School of Medicine, Chicago, IL USA; 8 Department of Pathology, Northwestern University Feinberg School of Medicine, 303 E. Chicago Ave, Chicago, IL 60611 USA

## Abstract

**Background:**

Initiating early effective antimicrobial therapy is the most important intervention demonstrated to decrease mortality in patients with gram-negative bacteremia with sepsis. Rapid MIC-based susceptibility results make it possible to optimize antimicrobial use through both escalation and de-escalation.

**Method:**

We prospectively evaluated the performance of the Accelerate Pheno™ system (AXDX) for identification and susceptibility testing of gram-negative species and compared the time to result between AXDX and routine standard of care (SOC) using 82 patient samples and 18 challenge organisms with various confirmed resistance mechanisms. The potential impact of AXDX on time to antimicrobial optimization was investigated with various simulated antimicrobial stewardship (ASTEW) intervention models.

**Results:**

The overall positive and negative percent agreement of AXDX for identification were 100 and 99.9%, respectively. Compared to VITEK® 2, the overall essential agreement was 96.1% and categorical agreement was 95.4%. No very major or major errors were detected. AXDX reduced the time to identification by an average of 11.8 h and time to susceptibility by an average of 36.7 h. In 27 patients evaluated for potential clinical impact of AXDX on antimicrobial optimization, 18 (67%) patients could potentially have had therapy optimized sooner with an average of 18.1 h reduction in time to optimal therapy.

**Conclusion:**

Utilization of AXDX coupled with simulated ASTEW intervention notification substantially shortened the time to potential antimicrobial optimization in this cohort of patients with gram-negative bacteremia. This improvement in time occurred when ASTEW support was limited to an 8-h coverage model.

## Background

Gram-negative bacteria account for up to half of all bloodstream infections (BSIs) in hospitalized patients, and the incidence is increasing for catheter-associated BSIs. The mortality rate of patients with gram-negative bacteremia ranges from 12 to 38%. Initiating early effective antimicrobial therapy is the most important intervention demonstrated to decrease mortality in patients with gram-negative bacteremia with sepsis [1–3]. On the other hand, overuse of broad-spectrum antimicrobial agents can increase unnecessary healthcare utilization, rates of iatrogenic infections such as *Clostridium difficile* colitis, and antimicrobial resistance [4]. Studies evaluating use of rapid diagnostic tests (RDTs) to target antimicrobial therapy have suggested improved time to appropriate antimicrobials and better outcomes [5].

Most currently available RDTs provide organism identification (ID) and/or genotypic resistance profiles [6, 7]. The AXDX (Accelerate Diagnostics, Inc., Tucson, AZ) utilizes fluorescence in situ hybridization technology to provide pathogen identification and morphokinetic cellular analyses to obtain phenotypic antimicrobial susceptibility results [8]. Compared to the detection of resistance markers offered by some RDTs for the diagnosis of bloodstream infections, MIC-based susceptibility results make it possible to optimize antimicrobial use through both escalation and de-escalation.

Previous literature has demonstrated that in patients with bloodstream infections, RDTs have minimal clinical impact without proper antimicrobial stewardship support [9, 10]. The goal of antimicrobial stewardship programs (ASPs) is to optimize antimicrobial use while minimizing its potential negative consequences, including antimicrobial resistance, adverse events, hospital-acquired infections, and added cost. Combining RDTs with different ASP supporting models has been explored [6].

The objectives of this study were (1) to evaluate the performance of AXDX for gram-negative bacterial ID and antimicrobial susceptibility testing (AST) compared with our standard laboratory procedure, and (2) to explore the potential benefit of utilizing AXDX in combination with various ASTEW intervention models on time to antimicrobial optimization in patients with gram-negative BSIs.

## Methods

### Ethics statement

This was a single-center study conducted at Northwestern Memorial Hospital, an 894-bed academic medical center located in Chicago, Illinois. Study samples were residual clinical specimens not specifically collected for study purposes. The study was approved by the Institutional Review Board (IRB) at Northwestern University.

#### Study samples and microbiology laboratory process

Blood culture was performed with the BacT/ALERT® system (bioMérieuxbioMérieux, Durham, NC). Each blood culture included one aerobic bottle and one anaerobic bottle which were incubated for up to five days until positivity. Only one blood culture from each patient was used in the study. Positive blood cultures were processed for ID and AST 24 h a day and 7 days a week. Eighty-two non-duplicate patient samples were included in the study. In addition, 18 challenge isolates with well-characterized resistance mechanisms were also used. The challenging isolates were diluted to 1 × 10^6^ CFU/ml with sterile saline and mixed with 10 ml of whole blood before inoculating into the blood culture bottle.

Once a blood culture flagged positive, Gram stain was performed followed by testing with the Accelerate PhenoTest™ BC kit within 8 h of growth detection. Only blood cultures positive for gram-negative bacilli were included in the study. The gram-negative testing panel includes *Pseudomonas aeruginosa, Acinetobacter baumannii, Klebsiella* spp. (i.e., *Klebsiella pneumoniae, Klebsiella oxytoca*, not differentiated), *Escherichia coli, Enterobacter* spp. (i.e., *Enterobacter cloacae, Enterobacter aerogenes,* not differentiated*), Proteus* spp. (i.e., *Proteus mirabilis, Proteus vulgaris,* not differentiated), *Citrobacter* spp. (i.e., *Citrobacter freundii, Citrobacter koseri,* not differentiated), and *Serratia marcescens.* Cultures positive for gram-negative rods were sub-cultured to a sheep’s blood agar plate (BAP), a chocolate agar plate (CHOC), and a MacConkey agar plate (MAC). For each sample, times were recorded for Gram stain notification, the AXDX ID and AST results, and the VITEK® MS ID and VITEK® 2 AST results as standard of care (SOC) via the electronic health record (EHR)**.** VITEK MS ID was performed three times during the first shift, and once each during second and third shifts whenever sufficient growth was detect on plate. AST with the VITEK® 2 GN70 was performed with overnight plate growth. Cultures with more than one organism were sub-cultured before VITEK 2 testing. AST was performed once a day during the first shift. Aliquots of blood culture samples were frozen for later adjudication testing. AXDX was performed right after the Gram stain result was obtained.

#### Discrepancy resolution

Culture was repeated with the frozen samples that showed identification discrepancies between AXDX and SOC. Frozen samples were plated on BAP, CHOC, and MAC plates, and incubated at 35 °C for 24 h. The frozen aliquot was also sent for third-party testing with the VITEK® 2 system. When third-party testing confirmed an AXDX positive result or an AXDX negative result, the AXDX result was reported as a true positive or true negative result, respectively. When third-party testing did not confirm an AXDX positive result or an AXDX negative result, the AXDX result was reported as a false positive or false negative result, respectively.

The frozen isolates recovered from samples with discrepant AST categorical results were tested in triplicate with standard broth microdilution (BMD) from Clinical Laboratory Standards Institute (CLSI) to obtain a modal MIC result. Frozen isolates were sub-cultured twice before AST testing.

#### Stewardship simulation

Twenty-seven adult patients (≥ 18 years old) with ≥1 positive blood culture containing a gram-negative rod isolate were enrolled from February 2017 to May 2017. Informed consent was obtained per IRB protocol.

Routine SOC and AXDX were performed following the process described above. Our current institutional laboratory protocol dictates that a physician from the primary treatment team must be notified with the Gram stain result via phone in less than 1 h after bacterial growth is detected by BacT/ALERT®.

A Simulated ASTEW intervention was performed after AST results for the both VITEK® 2 and AXDX were available. A 2-h response time was allotted for a stewardship intervention if the antimicrobials needed to be optimized and the stewardship team was onsite. This 2-h turn-around time corresponds to the time needed for a stewardship team member to review the patient chart, contact the primary team, have the order processed by the pharmacy, and have the new antimicrobial delivered to the patient’s nurse for administration.

Given that the results were reported 24 h per day, varying degrees of simulated stewardship coverage were explored to determine possible impacts on times to ASTEW; these included shift times of 8-h (0800–1600), 16-h (0800–0000), and 24-h coverage. If the AXDX results were reported during off-hours, then the time to ASTEW was recorded 2 h after the start of the next active shift. For example, if the AXDX results were recorded at 0200 for simulated 16-h stewardship coverage, then the time to antimicrobial optimization based on ASTEW would be recorded at 1000 (0800 + 2 h).

As the control, antimicrobial stewardship team notification is not routinely performed but team members are available on-site during daytime hours (0800–1630) Monday through Friday and via pager off-site all other times to answer questions and make recommendations when necessary. AXDX results were not available to the stewardship team.

#### Antimicrobial optimization

Data on both empiric and targeted antimicrobial therapies were recorded. An antimicrobial therapy was defined as optimal when it was the narrowest spectrum agent with acceptable activity against the isolated pathogen based on the AST results. Every case was reviewed by a panel of 3 infectious disease specialists from the ASTEW team (2 infectious disease pharmacists and 1 infectious disease physician) and 2 of the 3 specialists had to agree for the agent to be considered optimal therapy. The time to optimal antimicrobial therapy was recorded from the time of Gram-stain notification to the time that the first dose of optimal therapy was administered as noted on the electronic medical record. If the antimicrobial therapy was not optimal, the time to optimal therapy was recorded as the time the patient was discharged or when the antimicrobial regimen was completed, whichever was sooner.

#### Outcomes evaluation

The primary outcome measure was the simulated difference in time to antimicrobial optimization when utilizing AXDX with 8-h ASTEW coverage compared to our institutional SOC without ASTEW support. If antimicrobial optimization occurred prior to AXDX AST results, the time difference was recorded as zero. Secondary outcome measures included the simulated differences in time to antimicrobial optimization when utilizing AXDX with 8-h, 16-h, and 24-h ASTEW coverage compared to our institutional SOC with 8-h ASTEW coverage.

#### Statistical analysis

Positive percent agreement (PPA) and negative percent agreement (NPA) were calculated for each AXDX ID result compared to the SOC result. In addition, positive predictive value (PPV) was calculated for the AXDX monomicrobial calls compared to SOC.

Essential agreement (EA), categorical agreement (CA), very major error (VME), major error (ME), and minor error (mE) rates were calculated as a way to measure the AST accuracy of AXDX compared to those of the SOC for each antimicrobial tested. EA is the percentage of the total test results within one doubling dilution of the SOC result. CA is the percentage of the total test results with the same categorical interpretation result as the SOC result. VME is the percentage of the resistant isolates by the SOC that tested susceptible by the AXDX. ME is the percentage of the susceptible isolates by the SOC that tested resistant by the AXDX. mE is the percentage of the total test results in which one result (from the AXDX or the SOC) is intermediate and the other is not.

The time difference to antimicrobial optimization versus the SOC arms were analyzed by paired student t-tests. A one-way ANOVA was utilized to assess a difference in optimization time between the three arms of 8, 16, and 24-h stewardship coverage. Statistical analyses were performed using Microsoft Excel 2013 and SPSS Version 23.0 (IBM Corp., Armonk, NY).

## Results

### AXDX assay identification evaluation

Blood cultures from 82 patients and 18 challenge isolates were tested. Ten patient samples were excluded from the statistical analysis; reasons for exclusion include: (1) AXDX testing was not performed within 8 h of sample positivity (*n* = 4) as required by the package insert; (2) AXDX failed to complete the test (*n* = 2) due to technical issue; (3) AXDX failed to produce identification results for four samples. Two of them grew off-panel organisms (1 *Stenotrophomonas maltophilia*, 1 non-GNR *Streptococcus sanguinis*) and no growth was detected from blood culture bottle for the other two samples. The remaining 90 samples were used to calculate ID performance and included 72 fresh samples and 18 seeded samples.

Table 1 summarizes the ID performance after adjudication, and Table 2 summarizes the discrepant identification results. Before adjudication, four cultures had discrepant identification results by AXDX. One culture was positive for *K. pneumoniae* by SOC but positive for both *E. coli* and *Klebsiella* spp. by AXDX, which was confirmed by discrepancy testing. One culture tested positive by SOC for three organisms, *K. pneumoniae*, *K. oxytoca*, and *C. freundii*, but was only positive for *Klebsiella* spp. by AXDX. Adjudication testing confirmed *K. pneumoniae* and *K. oxytoca,* but not *C. freundii*. Therefore, the AXDX result was classified as true positive because AXDX *Klebsiella* spp. probe not does not differentiate between *K. pneumoniae* and *K. oxytoca.* Another culture tested positive for *E. coli* and *Enterococcus avium* by SOC, while AXDX only detected *E. coli*. Again, the AXDX result was classified as a true positive because *E. avium* is an off-panel organism for AXDX. The fourth discrepant result was reported as *Leclerica adecarboxylate,* by SOC which AXDX reported as *Enterobacter* spp.... Adjudication testing indicated *Leclerica adecarboxylate*, which was classified as a false positive result for AXDX. Thus, after adjudication, the overall PPA and NPA for AXDX compared to VITEK MS were 100 and 99.9%, respectively (Table 1). Out of 90 confirmed monomicrobial cultures, 70 cultures had a positive monomicrobial call by AXDX. No false positive monomicrobial calls were produced, yielding a PPV of 100%.

**Table 1:** Summary of identification results of AXDX after adjudication using SOC as the reference method

Organism	True Pos	False Pos	True Neg	False Neg	PPA	NPA
*Acinetobacter baumannii*	1	0	90	0	100%	100%
*Citrobacter* spp.	3	0	88	0	100%	100%
*Enterobacter* spp.	8	1	82	0	100%	98.8%
*Escherichia coli*	35	0	56	0	100%	100%
*Klebsiella* spp.	23	0	68	0	100%	100%
*Proteus* spp.	5	0	86	0	100%	100%
*Pseudomonas aeruginosa*	10	0	81	0	100%	100%
*Serratia marcescens*	4	0	87	0	100%	100%
All	89	1	638	0	100%	99.9%

*Pos* positive, *Neg* negative, *PPA* positive percent agreement, *NPA* negative percent agreement

**Table 2:** Discrepant identification results method

SOC Result	AXDX	3rd Party Testing Result
*K. pneumoniae*	*K. pneumoniae, E. coli*	*K. pneumoniae, E. coli*
*K. pneumoniae, K. oxytoca, C. freundii*	*K. pneumoniae*	*K. pneuminae, K. oxytoca*
*E. coli, E. avium*	*E. coli*	*E. avium* is not on AXD panel
*Leclercia adecarboxylata*	*Enterbacter* spp.	*Leclercia adecarboxylata*

*SOC* standard of care, *AXDX* Accelerate Pheno™ system

### AXDX AST results

AXDX AST results for 87 samples yielded an overall EA of 96.1% and CA of 95.4% after adjudication (Table 3). One VME for *E. coli* with ceftriaxone and two MEs (one for *E. coli* with aztreonam, and one for *K. pneumoniae* with ampicillin-sulbactam) were adjudicated to minor errors following discrepancy testing by BMD (Table 4). After adjudication, no VMEs or MEs were detected, but forty minor errors were detected: ampicillin-sulbactam (*n* = 14), tobramycin (*n* = 7), piperacillin-tazobactam (*n* = 5), and cefepime and ceftriaxone (*n* = 4 each), aztreonam (*n* = 3), meropenem (*n* = 2) and ertapenem (*n* = 1).

**Table 3:** Summary of the antimicrobial susceptibility testing results of AXDX, after adjudication

Antimicrobial	EA		CA		VME	ME	mE	S	I	R
	No. results/total	%	No. results/total	%						
Amikacin	82/82	100%	84/84	100%	0	0	0	77	0	7
Ampicillin-Sulbactam	57/62	91.9%	48/62	77.4%	0	0	14	30	7	25
Aztreonam	71/74	95.9%	72/75	96%	0	0	3	54	1	20
Cefepime	79/82	96.3%	79/83	95.2%	0	0	4	60	2	21
Ceftriaxone	74/74	100%	71/75	94.7%	0	0	4	52	1	22
Ciprofloxacin	79/83	95.2%	84/84	100%	0	0	0	48	0	36
Ertapenem	72/73	98.6%	72/73	98.6%	0	0	1	65	0	8
Gentamicin	80/83	96.4%	84/84	100%	0	0	0	66	0	18
Meropenem	79/83	95.2%	83/85	97.6%	0	0	2	70	1	14
Piperacillin-Tazobactam	63/68	92.6%	70/75	93.3%	0	0	5	55	3	17
Tobramycin	78/83	94%	77/84	91.7%	0	0	7	62	5	17
Overall	814/847	96.1%	824/864	95.4%	0	0	40	639	20	205

*EA* essential agreement, *CA* categorical agreement, *VME* very major error, *ME* major error, *mE* minor error, *S* susceptible, *I* intermediate, *R* resistant

**Table 4:** Discrepant susceptibility testing results

Organism	Antimicrobials	MIC			S/I/R			AXDX Error	
		AXDX	SOC	BMD	AXDX	SOC	BMD	vs. SOC	vs. BMD
*E. coli*	Ceftriaxone	1	≥64	2	S	R	I	VME	mE
*E. coli*	Aztreonam	16	4	8	R	S	I	ME	mE
*K. pneumoniae*	Ampicillin-Sulbactam	32	8	16	R	S	I	ME	mE

*MIC* minimal inhibitory concentration, *SIR* susceptible/intermediate/resistant, *VME* very major error, *ME* major error, *mE* minor error

### Time to results

Time to results for ID and AST by SOC and AXDX were evaluated, starting at the time of growth detection by BacT/ALERT®. Only data from fresh patient samples, and not the isolates, were included in this analysis. The average time for ID and AST results by SOC were 17.5 h and 47.8 h respectively (Table 5). The difference in the average time for ID by AXDX was 11.8 h sooner than SOC. The overall time for AXDX AST results was 36.7 h earlier than the result for SOC.

**Table 5:** Average times^a^ to ID and AST for AXDX versus SOC in hours

	AXDX (instrument time)	AXDX (time from positivity)	SOC (time from positivity)	Time Difference (SOC-AXDX)
ID	1.3	5.7	17.5	11.8
AST	6.6	11.1	47.8	36.7

^a^The average time for ID was calculated only with samples where ID probe results were in agreement between SOC and AXDX. The average time for AST was calculated only with samples where microbe and drug were both reported by SOC and AXDX

*SOC* standard of care, *AXDX* Accelerate Pheno™ system

### Potential impact of AXDX on patient antimicrobial management

Twenty-seven patients with GNR bloodstream isolates consented to participate in this study. From these patients, organisms identified included 9 (33%) *Klebsiella* spp., 6 (22%) *E. coli*, 4 (15%) *P. aeruginosa*, 3 (11%) *Enterobacter* spp., 2 (7%) *Citrobacter* spp., 2 (7%) *S. marcescens*, and 1 (4%) *Proteus* spp. Nine patients (33%) had antimicrobials administered that were considered optimal prior to AXDX results. Of the remaining 18 patients, 3 (11%) required escalation of therapy and 15 (56%) warranted de-escalation of therapy. The cases that required escalation of therapy included 1) a *K. pneumoniae* carbepenamase-producing isolate from a urinary source that required alteration of piperacillin-tazobactam therapy, 2) a multi-drug resistant *P. aeruginosa* secondary to an osteomyelitis that required alteration of cefepime therapy, and 3) a *K. pneumoniae* from a liver abscess with poor source control that warranted a switch from initial piperacillin-tazobactam therapy that had a MIC reported at the CLSI 2016 breakpoint of 16/4 μg/ml.

### Time for antimicrobial optimization with ASTEW intervention

Table 6 lists the time from blood culture order to AST results and time to antimicrobial optimization assuming 8-h ASTEW coverage. The mean improvement in time to potential antimicrobial optimization using AXDX with 8-h stewardship coverage was 18.1 h (95% CI, 11.6–24.2 h). When stewardship coverage was extended for AXDX testing from 8 h to 16 or 24 h, times to potential antimicrobial optimization were 22.6 h (95% CI, 15.8–29.4 h) and 23.2 h (95% CI, 16.5–29.9 h), respectively. The incremental improvements in time to antimicrobial optimization based on the varying degrees of ASTEW support were not statistically significant between groups (*p* = 0.443).

**Table 6:** Time to AST test results and the time for antimicrobial optimization for actual (SOC) and simulated (AXDX) testing methods

Testing Method	Time to AST Result (h)	Time for Antimicrobial Optimization (h)
Actual (SOC)	72.4 ± 16.9	54.7 ± 28.3
Simulated (AXDX)	35.9 ± 18.9	36.6 ± 24.0
Mean improvement	36.5	18.1

*AST* antimicrobial susceptibility testing

## Discussion

This study was limited to gram-negatives due to the greater potential for early optimization with gram-negative isolates. For the 8 g-negative target organisms evaluated in this study, the AXDX system yielded 100% PPA and 99.9% NPA for organism identification, and 96.1% EA and 95.4% CA for susceptibility results with no very major or major errors. Compared to our SOC procedure, AXDX would have potentially reduced time to ID by 11.8 h and time to AST by 36.7 h. In our study cohort, 66.7% of patients would have benefited from antimicrobial optimization guided by AXDX results. In the 8-h ASTEW coverage model, AXDX would have potentially improved time to antimicrobial optimization by 18.1 h.

The benefit of reducing the time to result for ID and AST was demonstrated in our study as well as in other studies [11]. However, our decrease in time to results was smaller than the results from other studies, especially for [12, 13]. This is likely due to the workflow difference among laboratories. In our lab, ID by VITEK® MS was performed five times a day throughout three shifts during each 24-h period. This workflow in our SOC arm minimized the waiting time for positive blood cultures.

The purpose of this study was to compare the AXDX technology to SOC methodologies for culture and susceptibility testing and to evaluate its impact on time to actionable ASTEW intervention.. Following the recognition that appropriate and timely antimicrobial therapy increased survival for patients with sepsis, the use of broad spectrum antimicrobials as the initial prophylactic treatment was widely adopted. Timely adjustment of the initial prophylactic treatment to targeted therapy is possible when rapid AST results are available. We showed that AXDX results, combined with stewardship notification, can result in substantially faster times to active therapy when escalation is needed, and to targeted therapy when de-escalation is warranted. In clinical practice, a greater than 1.5-day improvement in time to AST results and ASTEW could translate into significantly improved clinical outcomes, such as hospital length of stay and overall mortality as shown in previous publications [1, 14]. We also studied various ASTEW intervention models in an attempt to simulate real-world constraints on ASTEW resources. Analysis of different stewardship coverage models (excluding the addition of weekend days) showed only limited additional benefit when ASTEW coverage was extended from 8 h to 24 h, indicating that even institutions that have stewardship programs with limited resources can benefit from quicker ID and AST results. To combat issues such as broad-spectrum antimicrobial exposure and emergence of multi-drug resistant organisms, incorporating faster AST results from AXDX into patient management has the potential to improve individual patient outcomes and to assist broader stewardship efforts. There were multiple limitations of this study. The small sample size limits the generalizability of the results. It is unclear whether additional improvements in the time to antimicrobial optimization would have been observed if more samples would have been included. Our institutional laboratory SOC also limits the external validity of this study. MALDI-TOF and VITEK2, combined with batch testing and reporting, was used in this study; institutions with other testing or reporting methods will likely observe different results. Additionally, this was a non-interventional simulation study, so results in a measured clinical study may be more variable. Finally, the time improvements noted in our study were based on 100% acceptance of our stewardship team recommendations. In clinical practice, this is likely to be lower, which could further impact the magnitude of the results. The subjective nature of the stewardship team recommendations relative to what constitutes optimal antimicrobial therapy should also be taken into account.

## Conclusion

The utilization of AXDX coupled with simulated ASTEW notification substantially shortened the time to potential antimicrobial optimization in this cohort of patients with gram-negative bacteremia. This improvement in time occurred when ASTEW support was limited to an 8-h coverage model.

## Data Availability

The datasets generated during and/or analyzed during the current study are available from the corresponding author on reasonable request.

## Abbreviations

ASPs: Antimicrobial Stewardship Programs
AST: Antimicrobial Susceptibility Testing
ASTEW: Antimicrobial Stewardship
AXDX: Accelerate Pheno™ system
BSIs: Bloodstream Infections
CA: Categorical Agreement
EA: Essential Agreement
HER: Electronic Health Record
ME: Major Error
mE: Minor Error
NPA: Negative Percent Agreement
PPA: Positive Percent Agreement
RDTs: Rapid Diagnostic Tests
SOC: Standard of Care
VME: Very Major Error
